# High-quality genome-scale metabolic network reconstruction of probiotic bacterium *Escherichia coli* Nissle 1917

**DOI:** 10.1186/s12859-022-05108-9

**Published:** 2022-12-30

**Authors:** Max van ‘t Hof, Omkar S. Mohite, Jonathan M. Monk, Tilmann Weber, Bernhard O. Palsson, Morten O. A. Sommer

**Affiliations:** 1 The Novo Nordisk Foundation Center for Biosustainability, Technical University of Denmark, 2800 Kongens Lyngby, Denmark; 2 Department of Bioengineering, University of California San Diego, La Jolla, CA 92093 USA

**Keywords:** Genome-scale metabolic models, Probiotic, Nissle 1917, Phenotype microarray, Secondary metabolites, Constraint-based flux analysis

## Abstract

**Background:**

*Escherichia coli* Nissle 1917 (EcN) is a probiotic bacterium used to treat various gastrointestinal diseases. EcN is increasingly being used as a chassis for the engineering of advanced microbiome therapeutics. To aid in future engineering efforts, our aim was to construct an updated metabolic model of EcN with extended secondary metabolite representation.

**Results:**

An updated high-quality genome-scale metabolic model of EcN, iHM1533, was developed based on comparison with 55 *E. coli/Shigella* reference GEMs and manual curation, including expanded secondary metabolite pathways (enterobactin, salmochelins, aerobactin, yersiniabactin, and colibactin). The model was validated and improved using phenotype microarray data, resulting in an 82.3% accuracy in predicting growth phenotypes on various nutrition sources. Flux variability analysis with previously published ^13^C fluxomics data validated prediction of the internal central carbon fluxes. A standardised test suite called Memote assessed the quality of iHM1533 to have an overall score of 89%. The model was applied by using constraint-based flux analysis to predict targets for optimisation of secondary metabolite production. Modelling predicted design targets from across amino acid metabolism, carbon metabolism, and other subsystems that are common or unique for influencing the production of various secondary metabolites.

**Conclusion:**

iHM1533 represents a well-annotated metabolic model of EcN with extended secondary metabolite representation. Phenotype characterisation and the iHM1533 model provide a better understanding of the metabolic capabilities of EcN and will help future metabolic engineering efforts.

**Supplementary Information:**

The online version contains supplementary material available at 10.1186/s12859-022-05108-9.

## Background

Genome-scale metabolic models (GEMs) are becoming an increasingly popular and useful tool to understand and engineer microbial metabolism. The first GEM of *Escherichia coli* was reconstructed in the early 2000s, and has subsequently been updated with various biological functions and improved prediction capabilities [1, 2]. The latest GEM of *Escherichia coli* K-12 MG1655, iML1515, represented the knowledge base of that strain’s metabolism at that time, and is used frequently in metabolic engineering applications for the production of desired chemicals [1]. More recently, strain-specific GEMs of several species have been developed to investigate metabolic diversity across specific strains within species [3, 4, 5]. For example, GEMs of 55 fully sequenced *Escherichia* and *Shigella* strains were previously reconstructed to investigate functional differences between strains [3]. Further, a GEM for *E. coli* BL21(DE3), a common industrial microbe, was recently developed for biotechnological applications [6]. Reconstructing high-quality GEMs of other commonly used *E. coli* strains will further enhance the application of GEMs and constraint-based modelling tools for engineering purposes.

*Escherichia coli* Nissle 1917 is a gram-negative probiotic strain commonly used for metabolic engineering and synthetic biology applications. *E. coli* Nissle 1917 (also referred to as strain DSM 6601, O6:K5:H1, EcN) was detected and isolated in 1917 by Alfred Nissle due to its antagonistic activity against pathogenic *Salmonella* strains [7]. Historically, EcN has been used in its native form to treat various gastrointestinal conditions. EcN is sold commercially as Mutaflor and is clinically approved to treat diseases such as inflammatory bowel disease and ulcerative colitis [8, 9]. Over the last few years, the strain has been engineered for the expression of therapeutic activities to treat a wider range of diseases, both inside and outside of the gut [10, 11, 12]. EcN belongs to the *E. coli* phylogenetic group B2 that includes well-known clinical pathogens such as *E. coli* CFT073. However, EcN is non-invasive, lacks most known virulence factors like α-hemolysin and P/M/S-fimbriae, does not produce most toxins associated with other pathogenic *E. coli* strains, is serum-sensitive, and is not resistant to commonly used antibiotics [13]. EcN carries various genomic islands (GEIs), which include genes encoding for several fitness factors, like microcins which exhibit antibacterial activity, but also pathogenicity islands, like *pks. *The *pks* island is associated with EcN’s probiotic effect, but its product, the genotoxin colibactin, has been linked to colorectal cancer [14, 15, 16, 17]. Additionally, the siderophores produced from other genomic islands provide versatile iron transport systems assisting EcN’s survival in the gut by competing with pathogenic enterobacteria [18]. For example, yersiniabactin allows EcN to acquire zinc in the inflamed gut to compete with pathogenic *Salmonella* strains [19]. Overall, the specific phenotype of EcN in combination with the availability of well-developed synthetic biology tools for *E. coli* strains makes it a desirable candidate for cell-factory design and engineering.

GEMs computationally describe properties of a strain, enable the prediction of metabolic fluxes at various conditions, and provide gene manipulation targets for metabolic engineering. Additionally, GEMs offer a platform to integrate and analyse multi-omics and kinetic datasets [20]. The complete genome for EcN was first sequenced in 2014 and resequenced multiple times afterwards [21]. Recent years have also seen the generation of transcriptomics [22, 23], metabolomics [24], and ^13^C metabolic flux data [25] of EcN. The advances in omics data generation of EcN and its unique metabolic capabilities demand a high-quality GEM for EcN that will help in diverse engineering applications in the future.

A GEM of EcN called iDK1463 was published based on the first genome assembly of EcN (CP007799.1) [26]. Building on this initial model we created an updated GEM for EcN called iHM1533, which was validated using phenotypic microarray data and ^13^C fluxomics. The final model was used to investigate engineering targets for optimisation of secondary metabolite production.

## Results

### Reconstruction of a draft genome-scale metabolic model for *E. coli* Nissle 1917

**Fig. 1:**
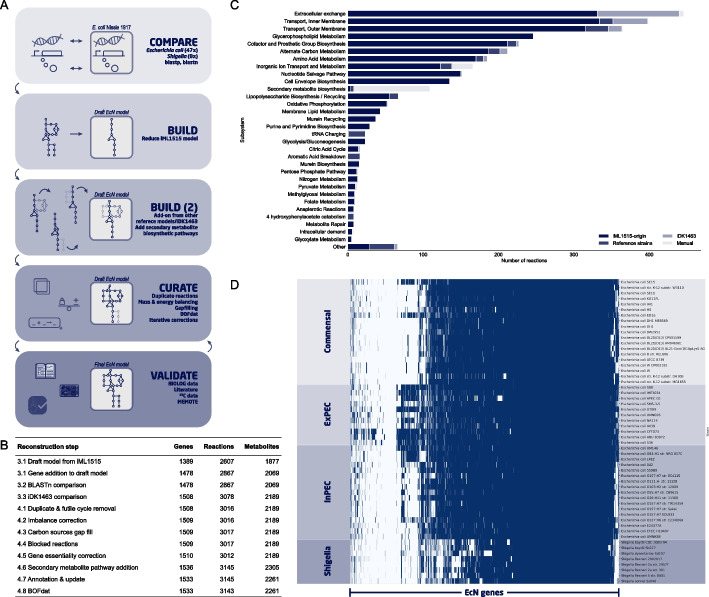
Genome-scale reconstruction steps and gene comparison. **A** Workflow of the construction of iHM1533 **B** All stages of the iHM1533 model construction, with the number of genes, reactions, and metabolites present in the model at each stage. **C** Origin of all reactions in iHM1533, divided by subsystem. The iHM1533 model is based on a reduced iML1515 model (iML1515-origin), to which reactions were added from 54 models of *E. coli*/*Shigella* strains (Reference strains) and the existing EcN model (iDK1463). During curation, additional reactions were added to correct the model and to expand various secondary metabolite biosynthesis pathways (Manual). **D** Heatmap of gene homology of all 55 strains to EcN. Strains were clustered in four groups: commensal, extraintestinal pathogenic *E. coli* (ExPEC), intestinal pathogenic *E. coli* (InPEC), and *Shigella*. Genes with homology above 80% were deemed homologous (blue) and those below 80% were not (white). The highest number of homologous genes is shared with ExPEC strains, which also has previously been observed by Grozdanov et al*.* [34], when comparing EcN to 324 non-pathogenic and pathogenic *E. coli* isolates

The GEM reconstruction process involved multiple steps based on comparative genomics, manual curation, and validation following published reconstruction protocols [5, 27] (Fig. 1A). A high-quality genome (CP022686.1 from 2018, as compared to CP007799.1 from 2014 used by Kim et. al.) of EcN was used for reconstruction, with a length of 5,055,316 bp and annotated with a total of 5045 genes. Additionally, EcN harbours two plasmids, pMUT1 and pMUT2 [28]. First, we compared the genome of EcN against 55 related strains with high-quality GEMs available, herafter referred to as reference strains, using bidirectional best blastp hits [3]. Of the 5045 EcN genes compared, 1783 were common among all strains and 196 were unique to EcN (Fig. 1D, Additional file 1: Data S1). We observed that the genome of EcN shared the highest number of homologous genes with uropathogenic *E. coli* strains (UPEC) such as CFT073 (87.0% of EcN genes) and ABU 83972 (86.8% of EcN genes), with which it may share a common ancestor [29, 30, 31, 32]. CFT073 has hitherto repeatedly been compared to EcN and ABU 83972, to identify potential causes for the difference in pathogenicity between these highly related strains [24, 29, 33]. The commonly used strain *E. coli* K-12 MG1655 has 3693 (73.2%) homologous genes. Several horizontally acquired DNA regions that are, at least partially, shared with other *E. coli* strains such as CFT073, are not present in MG1655. These regions may contribute to the difference in phenotypic traits of EcN [34]. EcN contains regions that are mostly shared with extraintestinal pathogenic *E. coli* (ExPEC) strains (Fig. 1D). EcN specific sequences (selected regions among genetic islands I to IV) were previously observed to be shared among many ExPEC strains, but less frequently observed in intestinal pathogenic *E. coli* (InPEC) and non-pathogenic strains [34]. The lowest number of homologous genes was shared with all *Shigella* strains (59.0–66.5%).

Using the gene homology analysis between EcN and all reference strains, a draft GEM was reconstructed based on the corresponding reference GEMs. This draft model consisted of 1487 genes, 2867 reactions, and 2069 metabolites (Fig. 1B). Of these reactions, 90.9% (2607) originated from the *E. coli* K-12 MG1655 model iML1515, which was used as the base for the construction of this model as this is one of the most highly curated *E. coli* models. One of the subsystems of which multiple reactions were added from the other reference strains was tRNA charging (22 reactions). When comparing the sequence context of tRNA loci in EcN versus K-12, 15 out of 37 tRNA loci varied due to the integration of horizontally acquired genetic information, including *asnT, argW, ileY, leuX, pheV, serX,* and *thrW* [34]. The genes encoding for these seven tRNA synthetases, among others, did not have homology with the K-12 genome and were added from models other than iML1515 during the construction of the draft model (Fig. 1C). The remaining 260 reactions added from other models belonged to subsystems such as various forms of transport systems (65 reactions), alternate carbon metabolism (16), and metabolism of various amino acids (Fig. 1C). Several of the 260 reactions were unassigned (26) or removed during manual curation (63).

Furthermore, an additional comparison between nucleotide sequences of all EcN genes (BLASTn) did not identify any additional genes to be added. Finally, the draft EcN model was compared to iDK1463 and 211 reactions only present in the latter were imported along with 30 additional genes (Fig. 1C, Additional file 2: Data S2) [26].

### Manual curation of EcN draft model to remove network inconsistencies

A thorough manual curation of the draft model was performed in multiple stages, which included removing inconsistencies, correcting mass and charge balances, gap filling with phenotype microarray data, and expansion of secondary metabolite biosynthetic pathways (Fig. 1B). In general, the reconstruction process followed the standard protocols [5, 27]. First, 48 reaction duplications caused by differences in directionality, metabolites, and gene rules across reference models were removed (e.g., MTHFD and MTHFD_1). Additionally, 14 reactions causing futile energy-generating cycles were corrected. Subsequently, mass and charge imbalances were identified and 53 such inconsistencies were manually resolved (6 remaining). One of the cases involved a mass imbalance of two reactions (ACGAL6PI & ACGAL6PISO) involving the metabolite N-acetyl-D-galactosamine-6-phosphate (GalNac-6-P). The conversion of GalNAc-6-P to Tag-6-P is performed in two steps: deacetylation of GalNAc-6-P to GalN-6-P and deamination/isomerisation of GalN-6-P to Tag-6-P [35]. Both original reactions were replaced by two reactions: ACGAL6PDA (deacetylation step) and GALAM6PISO (deamination/isomerisation step) with gene rules *agaA* and *agaS*, respectively (Additional file 3: Fig S1).

In the next stage of gap filling, we used an algorithm available in cobrapy [36] that evaluates a minimal set of reactions required to be added to make a pathway feasible [37]. All blocked pathways in the model were checked for gap filling using the panreactome of all the reference GEMs. Gap filling suggested that a malate decarboxylating oxidoreductase (‘MALDDH’) reaction was missing in the model, preventing growth on D-malate. Upon addition of this reaction, growth was restored in the model. However, no homologous gene could be identified in EcN that could be linked to this reaction. Presence of this ability without an identifiable genomic background gives the opportunity to discover new genes related to D-malate metabolism [38].

The previous genome-scale reconstructions of *E. coli* strains contained biosynthetic pathways for secondary metabolites like enterobactin. EcN produces a wider range of secondary metabolites that also includes salmochelins (glucosylated enterobactin), yersiniabactin, aerobactin, and colibactin (Additional file 3: Fig S2) [15]. Here, we expanded the representation of biosynthetic pathways for enterobactin, salmochelins, aerobactin, yersiniabactin, and colibactin. This process involved defining new reactions representing various biochemical steps common to non-ribosomal peptide synthetase (NRPS) and polyketide synthase (PKS) assembly, similar to the descriptions in the GEM of *Streptomyces coelicolor* [39] (Additional file 3: Fig S2). The various chemical steps involved in these pathways were added from available pathways for enterobactin, colibactin, salmochelins, and aerobactin in the MetaCyc database. In the case of yersiniabactin, however, the entire pathway was reconstructed using the literature [40]. In addition to biosynthetic reactions, we also included reactions involved in the transport of secondary metabolites and various metal ion import reactions related to them. In total, iHM1533 included 177 curated reactions and 110 metabolites to represent secondary metabolism of EcN (Additional file 2: Data S2).

Next, all metabolites, reactions, and genes were annotated with identifiers from external databases, such as KEGG, BioCyc, MetaNetX, SEED, and CHEBI. Inclusion of these identifiers is important for interoperability between various knowledge bases and multiple omics data types. The locus tag, gene ID, and protein ID were added for all genes, when available in the genbank file, and all disconnected genes and metabolites were removed from the model. Lastly, the biomass objective function was updated using BOFdat (Biomass Objective Function from experimental data) [41]. In the first step, the stoichiometric coefficients for the major macromolecules were defined. Genomic (CP022686.1), transcriptomic [42], and growth data [26] of EcN was used, while MG1655 data was used for the macromolecular composition, proteomics, lipidomics, and maintenance [41], as EcN data was lacking. Coenzymes and inorganic ions were identified in the EcN model and included in the biomass objective function in the second step. The newly defined function was used to update the existing iDK1643 biomass objective function and get the final GEM iHM1533 (Additional file 4).

The curated GEM restored the in silico growth of 1.05 h^–1^ on synthetic minimal medium with 15 mM glucose as a carbon source (when using iDK1463 and iML1515 respectively 1.07 and 1.33 h^–1^). The predicted growth rate is higher than the 0.79 ± 0.02 h^–1^ described in literature on this media [25]. Although the biomass function was adjusted using BOFdat, additional experimental data of EcN is required to further increase accuracy. Additionally, various factors such as constraints on total protein, catalytic rates, and transcriptional regulation were not included in the model.

### Phenotype characterisation of EcN

**Fig. 2:**
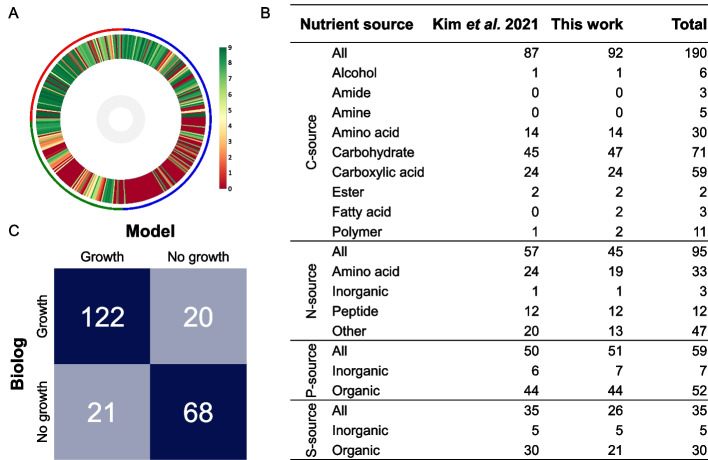
Phenotype characterisation of EcN and validation of GEM. **A** Circular heatmap of phenotype microarray data. PM1 and PM2 contain carbon sources (blue ring), PM3 nitrogen sources (green ring), and PM4 phosphorus and sulfur (red ring). For each nutrient, an activity index was assigned between 0 (no growth, red) and 9 (maximum growth, green). Activities of 3 and above were considered as growth. **B** Comparison of the number of nutrients that enabled the growth of EcN in the work of Kim et al*.* [26] and in this work. The carbon (C) source when testing the nitrogen (N)/phosphorus (P)/sulfur (S) source was succinate/citrate and pyruvate, respectively. Total is the total number of sources tested for each nutrient. **C** Comparison of phenotype microarray data of EcN (Biolog) to growth predictions made by the final iHM1533 GEM (Model)

EcN naturally resides in the large intestine. Successful colonisation of the gut environment is dependent on various factors, including nutrient specificity and efficiency. Therefore, we were interested in the range of nutrients EcN can metabolise. Here, we generated phenotype microarray data for this strain. Four different Biolog plates were used, PM 1-4, consisting of 190 carbon, 95 nitrogen, 59 phosphorus, and 35 sulfur nutrient sources. We analysed the phenotype microarray data using DuctApe software that assigns activity indices between 0 and 9 to represent growth on each nutrient [41]. An activity index of 3 and above was considered as growth. We found that EcN could utilise 92 of the 190 carbon sources that included 47 carbohydrates (of 71 total), 24 carboxylic acids (59 total), 14 amino acids (30 total), and others (Fig. 2A/B, Additional file 5: Data S3). For the nitrogen sources, 45 nutrients facilitated growth of EcN that included 19 amino acids (33 total), all 12 of the peptides, and others. EcN could utilise 51 of the 59 phosphorus nutrients including all 7 inorganic and 44 organic sources (52 total). EcN was also capable of growth on all 5 of the inorganic and 21 of the 30 organic sulfur nutrient sources.

Overall, EcN has a wide range of nutrient sources it can utilise. In the large intestine, various nutrients are supplied by ingested food, epithelial and bacterial cell debris, and the mucus lining of the epithelium. This includes, e.g., gluconate from muscle tissues and sugar acids like galacturonate from pectin [42]. The mucus lining is a combination of glycoproteins and glycolipids that provide nutrients to intestinal bacteria, including N-acetylglucosamine, N-acetylgalactosamine, galactose, fucose, sialic acid (N-acetylneuraminic acid), and lesser amounts of glucuronate and galacturonate [43]. EcN is capable of growing on all these compounds as a carbon source (Additional file 5: Data S3). The capacity of EcN to metabolise this range of carbohydrates makes sense when put into perspective with its origin in the gut.

We compared our dataset with previously published Biolog data of EcN (Fig. 2B) [26]. In our experiment, EcN was capable of growth on six carbon sources (adonitol, dulcitol, pectin, Tween 20, Tween 40, and a-hydroxybutyric acid) that did not permit growth of EcN in the published dataset (Additional file 5: Data S3). However, the latter could grow on b-hydroxybutyric acid, which was not the case in our work. For the non-carbon source profiling PM plates, a different carbon source was used in the published experiment, 2 M succinate/200 µM citrate, compared to 2 M pyruvate used in our experiment. On succinate, EcN was capable of metabolising 57 nitrogen, 50 phosphorus, and 35 sulfur nutrient sources. In comparison, EcN could metabolise on pyruvate 45 nitrogen (40 shared, 5 only with pyruvate, 17 only with succinate), 51 phosphorus (49 shared, 2 on pyruvate, 1 on succinate), and 26 sulfur (17 shared, 9 only with succinate) sources (Additional file 5: Data S3). We note that the comparison of growth prediction here against Kim et al. might also be affected by the cut-off of the activity index used for what is considered growth. Overall, we found that EcN can consume a wide range of nutrients of which many can be found in the gut and that utilisation of nitrogen and sulfur sources specifically can be dependent on the carbon source. This dataset can help with understanding EcN’s role in the gut microbiome and aid future genome engineering endeavours.

### Phenotypic characterisation of EcN largely corresponded to iHM1533 model predictions

Next, the phenotype microarray data was compared with growth predictions by iHM1533 to validate the accuracy of the reconstructed model (Fig. 2C). A total of 231 nutrient sources out of 379 on the Biolog plates could be linked to exchange reactions present in the model. EcN was able to grow on 20 nutrients that did not allow growth in the model (Additional file 5: Data S3). Gap filling was used to identify reactions that would enable the model to grow on these compounds from the panreactome of the reference strains, but no reactions were found that could restore growth [37]. For several pathways, e.g., methionine and phenylalanine metabolism, reactions had previously been added from the *E. coli* reference strains to the model (Fig. 1C). However, the pathways needed for growth on these compounds are not present in any of the models upon which iHM1533 was built, as none of them can grow on these compounds themselves [3]. As a result, these pathways could not be complemented by the reference stain panreactome. The model predicted growth on sucrose, while Biolog data showed the opposite. As EcN does not contain the genes for sucrose catabolism, the reactions SUCtpp, SUCptspp, SUCR, and FFSD were removed from the model [44].

Similar to iDK1463, reactions that are reported to be inactive under non-stressful aerobic conditions (ARGAGMt7pp, CELBpts, CLBtex, DTARTD, LCARS, PTRCORNt7pp, SUCTARTtpp, TARTD, and TARTRt7pp) were deactivated by constraining the flux to zero for this analysis [26]. Additionally, the catabolism of 3-hydroxyphenylacetic acid (3HPA) and 4-hydroxyphenylacetatic acid (4HPA) was deactivated by restricting flow through the reactions HPAtex and 3hoxpactex. Based on homology, genes required for the metabolism of these aromatic compounds are present in EcN, but Biolog data showed an inability of growth in normal aerobic conditions [45].

With the corrected model, growth on a total of 82.3% of nutrients was predicted correctly, which is just above the score of iDK1463 (81.8%) and in line with predictions in other recently published models (68–84%) [6, 46, 47]. We found 122 true positives and 68 true negatives, while 21 nutrients were incorrectly predicted to accommodate growth by the model and growth on 20 nutrients was still not supported in the model (Additional file 3: Table S1). Adonital and pectin, which only sustained growth in our Biolog dataset but not in the previously reported work [26], were among the 20 false-negative predictions. The false-positives included five nitrogen sources that showed very slow growth with an activity between 1 and 2.5 in the Biolog data set (l-Tyrosine, N-Acetyl-d-Glucosamine, N-Acetyl-d-Galactosamine, N-Acetyl-d-Mannosamine, and allantoin). All five sources did enable growth when succinate was used as a carbon source instead of pyruvate (Additional file 5: Data S3). Generally, false-positive predictions may occur if an enzyme is transcriptionally repressed, which is not represented in a metabolic model. Alternatively, an enzyme does not catalyse the designated reaction at a high enough rate under specific growth conditions and is thereby observed as false-positive, as appears to be the case for these five sources [48]. In addition to the Biolog data, growth on various carbon sources described in literature was checked [13, 25, 49]. iHM1533 correctly predicted growth on all 21 carbon sources, including l-arabinose, l-fucose, d-galactose, d-gluconate, and d-mannose (Additional file 6: Data S4). Therefore, the iHM1533 model could correctly predict a large part of the nutrient sustaining growth correctly, which is a prerequisite for making good predictions on behaviour in the gut or as a cell factory.

### ^13^C-fluxomics data comparison of EcN to iHM1533 model flux predictions

**Fig. 3:**
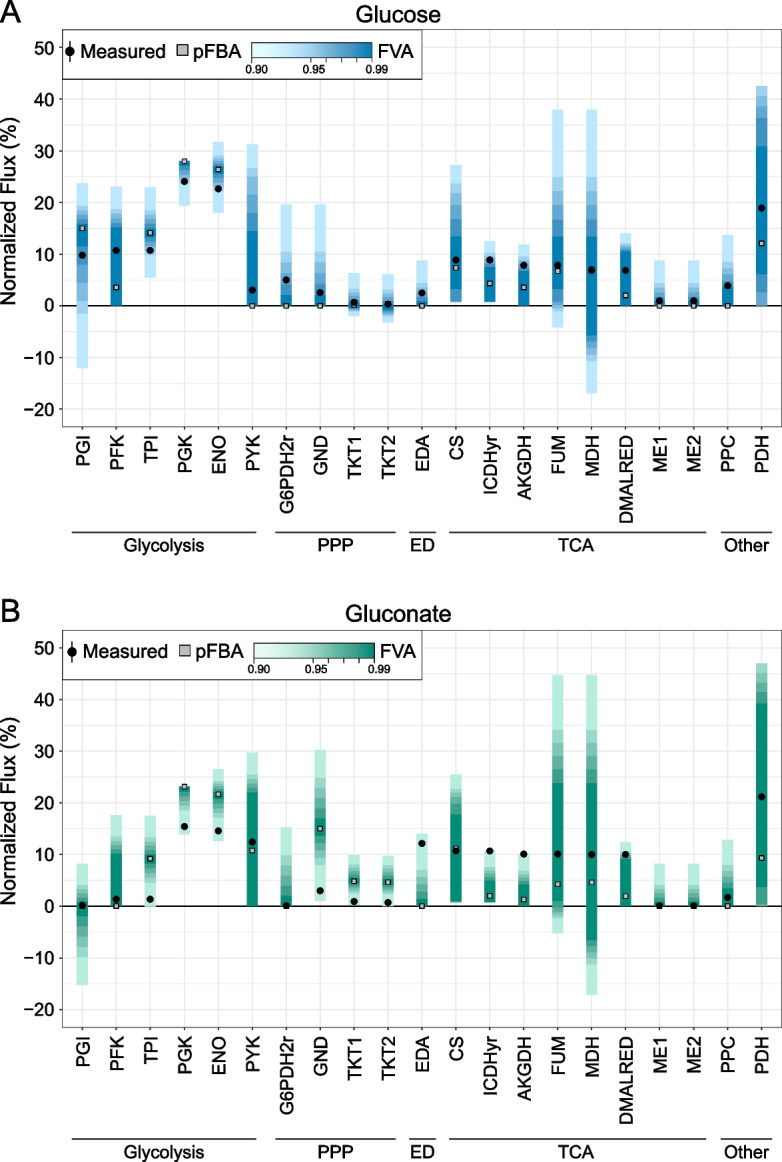
Comparison of ^13^C fluxomics data to iHM1533 flux predictions on minimal media. Comparison of predicted internal fluxes to experimental ^13^C fluxomics data from Revelles et al*.* [25] of EcN on glucose (**A**) and gluconate (**B**). The Flux Variability Analysis (FVA) was run with FVA thresholds from 90 to 99%. The Parsimonious FBA (pFBA) is depicted as squares and the experimental data is visualised with the mean (dots) and standard deviation of three biological replicates (error bars, too small to observe) The flux is normalised to the glucose/gluconate uptake rate. The conversion of malate to oxaloacetate can both be performed by malate dehydrogenase (MDH) and by malate:FAD oxidoreductase (DMALRED) that uses FAD^+^ instead of NAD^+^. Therefore both reactions are included

After comparing the GEM predictions with the Biolog data, the predictive quality of internal fluxes was validated with previously published ^13^C-fluxomics data. Fluxes determined by ^13^C experiments with growth on glucose and gluconate were compared to model-predicted fluxes [25]. The media conditions and efflux of acetate were set and internal fluxes were predicted in a sensitivity analysis with flux variability analysis (FVA) in combination with a Parsimoneous enzyme usage Flux Balance Analysis (pFBA). The predicted in silico fluxes and pFBA results were mapped to the experimentally determined fluxes on glucose and gluconate (Fig. 3, Additional file 7: Data S5). The pFBA shows limitations of predicting the fluxes in the tested conditions, for example a higher flux on glucose in the glycolysis (PGI) and a lower flux in the PPP (G6PDH2r). However, when the constraints in the objective function optimisation (biomass formation) were relaxed in the FVA, the internal central carbon flux corresponds more accurately to the experimental data. All experimental data points were within the 90% FVA threshold, and a large part within > 97%. These analyses validate the predictive quality of the model and display its use in prediction of internal fluxes based on media composition.

### Essential gene prediction of EcN in gut microbiome media using iHM1533

GEMs can be used to predict essential genes. iHM1533 predicted 156 genes and 230 reactions to be essential in anaerobic gut microbiome media (Additional file 8: Data S6). The reactions that were essential were linked, among others, to cofactor and prosthetic group biosynthesis (53), amino acid metabolism (66), and purine and pyrimidine biosynthesis (17). When compared to *E. coli* K-12 MG1655, one gene was found to be only essential in MG1655, *zupT*, and three were found to be only essential in EcN, *pgsA*, *can* and *leuB*. The latter two have been experimentally validated under aerobic conditions [26]. In Kim et al*.* (2021) the gene *argF* was additionally mentioned to be only essential in EcN, due to the lack of the gene *argI* in the genome. However we identified two genes in EcN encoding for an ornithine carbamoyltransferase [24, 50]. Both are annotated in the EcN genome as *argF,* with locus tags CIW80_16625 and CIW80_16605. During construction, only the first gene was included as *argF*. Therefore, the second locus tag was included in the model as *argI* and linked to the same reaction (OCBT).

In addition to single essential genes, we also investigated synthetic lethality. Synthetic lethality arises when knocking out a combination of two or more genes results in cell death while knocking out the individual genes does not. The predictions of synthetic double lethals encompassed a total of 158 gene combinations in aerobic conditions and 157 in anaerobic conditions (Additional file 8: Data S6). The difference was the combination of *hemN* and *hemF,* two oxidases catalysing the same step in heme biosynthesis. The knockout of *hemN* is lethal in anaerobic conditions, as the product of *hemF* is oxygen-dependent. In aerobic conditions this knockout is not lethal, except when combined with a *hemF* knockout [51]. Overall, the essential genes differ very little between anaerobic and aerobic conditions. Predictions of these essential genes could aid with and be targets in genome engineering studies in the future, for example in knockout studies or biocontainment strategies.

### Quality assessment of iHM1533 using Memote test suite

The final product, iHM1533, was tested using the Memote web application, a standard in the community for metabolic model testing [52] (Additional file 3: Fig S3). The analysis showed an overall score of 89%, which is a combination of the score on consistency and metabolite, reaction, gene, and SBO annotation. The consistency score was 97%. The score was lower than for iML1515, because in the secondary metabolite pathways of EcN a rest group (R) was added to multiple metabolites to represent the protein domains bound to the metabolite, resulting in mass and charge imbalances in iHM1533. Metabolite and reaction annotations scores were 84% and 83%, respectively. Nearly all genes have NCBI identifiers, but lack some other annotations (refseq, kegg, ccds, and hprd), resulting in a score of 68%. SBO terms had a score of 86%.

Further, we compared the Memote score of iHM1533 and iDK1463. The consistency score is the same, as an improvement in this score by the manual curation of the mass and charge balance in iHM1533 is negatively compensated by the imbalances resulting from the rest groups in the secondary metabolites. The annotations added as the last step of the curation resulted in a higher score for iHM1533 in all metabolite, reaction, and gene annotation scores. The SBO annotation was not updated and thereby gave a lower score for iHM1533. Overall the total score is the same, as the SBO score counts double.

### Prediction of manipulation targets for secondary metabolites produced by EcN

The updated and validated GEM was used to investigate relationships between primary and secondary metabolic pathways and predict engineering targets for the over-production of secondary metabolites. The parsimonious flux balance analysis showed that iHM1533 can produce all secondary metabolites, except for aerobactin under anaerobic gut microbiome media. The first step of aerobactin biosynthesis involved oxygenation of lysine and thus required oxygen (MetCyc: META:1.14.13.59-RXN) [53, 54]. Therefore we used gut microbiome media with oxygen in order to analyse all secondary metabolite fluxes.

**Fig. 4:**
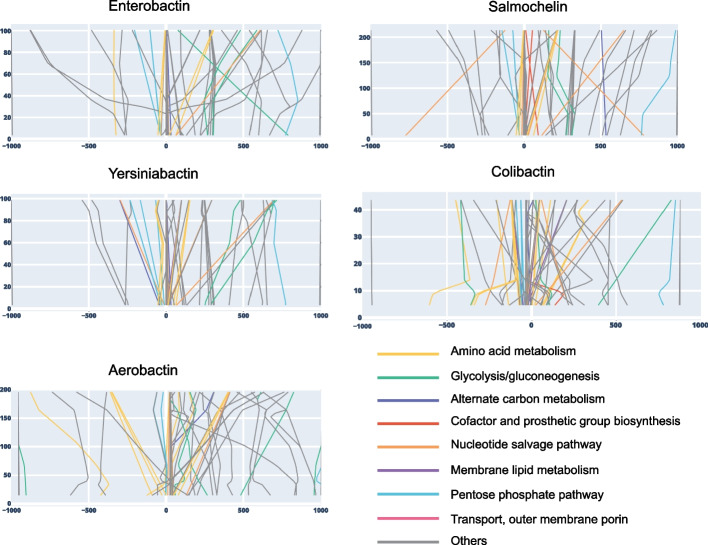
Predicted target reactions for secondary metabolite production using FSEOF. Line plots of variation of flux through secondary metabolite exchange reactions against enforced fluxes through predicted target reactions from various reaction subsystems (displayed by colour). A few membrane transport reactions of common metabolites such as H2Otpp, CO2tex, CO2tpp, H2Otex, and ATPS4rpp were ignored in the plots due to their large scale impact (Additional file 9: Data S7)

An algorithm called flux scanning with enforced objective function (FSEOF) was applied to identify targets for enhanced secondary metabolite production, while also allowing flux through biomass objective function [55]. The predicted targets from FSEOF analysis included reactions from various primary metabolism subsystems such as glycolysis/gluconeogenesis, various amino acid pathways, nucleotide salvage pathway, alternate carbon metabolism, and cofactor and prosthetic group biosynthesis, among others (Fig. 4, Additional file 3: Table S2). A combined total of 219 reactions that influence secondary metabolite production were predicted (Additional file 9: Data S7). Only 29 of 219 reactions were predicted as common targets for all secondary metabolites with 7 of them from the pentose phosphate pathway subsystem. On the other hand, 98 targets were predicted for only one of the secondary metabolites, including 40 for colibactin (24 of these from membrane lipid metabolism and 3 from methionine metabolism); 10 for salmochelin (3 of these from cofactor and prosthetic group biosynthesis); 30 for aerobactin (7 of these from threonine and lysine metabolism, where lysine is a key substrate for aerobactin); 9 for yersiniabactin (4 of these from methionine metabolism); and 9 for enterobactin (4 of these from nucleotide salvage pathway).

Further, we calculated the slope of flux variation of secondary metabolites against predicted FSEOF target reaction. A positive slope denotes increased flux through secondary metabolite as the flux through target reaction is increased and a negative slope denotes a decrease in secondary metabolite flux as the flux through target reaction is increased (Fig. 4, Additional file 9: Data S7). Reactions such as FBA, PFK, DHORDfum, and SUCDi were among the largest negative slopes common for all secondary metabolites, whereas PPA was one of the few reactions with a positive slope. Although many reactions for cofactor and prosthetic group biosynthesis were predicted as targets, the value of their slope was very low, indicating a minor impact on secondary metabolite biosynthesis. A total of 43 reactions from 10 different amino acid metabolism subsystems were part of the predicted target with mostly positive slopes. Reactions from cysteine and methionine metabolism were largely predicted as targets for yersiniabactin and colibactin biosynthesis. Reactions from tyrosine, tryptophan, and phenylalanine metabolism were predicted as targets for yersiniabactin, enterobactin, and salmochelin biosynthesis. Reactions from threonine and lysine metabolism were predicted specifically for aerobactin, whereas reactions from glycine and serine metabolism were largely predicted for all secondary metabolites but aerobactin. In general, these predictions of targets align with the amino acid substrates required as precursors for respective secondary metabolites.

Lastly, we carried out a biomass sensitivity analysis to measure the impact of the transition from primary to secondary metabolism across the subsystems. For this purpose, we created series of conditions where biomass reaction bounds were constrained to series of values between 0 and 100% of the optimal flux, while keeping the secondary metabolite exchange reaction as the objective function. This allowed us to measure reaction fluxes across all reactions at various stages going from no secondary metabolite production (100% biomasss) to maximum secondary metabolite production (0% biomass). The theoretically allowed flux ranges for all reactions at each biomass constraint were calculated using FVA, whereas the optimal solution flux was calculated using pFBA (Additional file 10: Data S8). To visualize the impact of primary to secondary metabolism, we visualised 10 reactions with the lowest slopes and 10 reactions with the highest slopes in the FSEOF analysis for each secondary metabolite (Additional file 3: Fig S4-S8). For example, there were a total of 9 targets predicted for improving aerobactin production from the subsystem of threonine and and lysine metabolism. Lysine is the key amino acid used as a precursor in aerobactin biosynthetic pathway. The predicted fluxes through reactions from threonine and lysine metabolism such as DAPE, SDPDS, DHDPRy, DAPDC increased signifincantly as the model was being optimised for aerobactin production over biomass, whereas the fluxes through SDPTA and ASAD from the same subsystem kept decreasing (Additional file 3: Fig S6). Our analysis thus shows the impact on the fluxes at systems level when the cell shifts from biomass to secondary metabolite production. In summary, this model can be used to investigate the link between primary and secondary metabolism and reactions can be predicted that provide targets for manipulation of secondary metabolites production.

## Discussion

*E. coli* Nissle 1917 has been used for many years in clinical settings and could prove to be a good chassis for advanced microbiome therapeutics in the future [11, 50, 56, 57, 58, 59, 60, 61]. Genome-scale metabolic models can aid in the understanding and engineering of EcN. To our knowledge, this was the first time a large number of multiple high-quality GEMs was combined to reconstruct a model. Even though the complete panreactome of 55 GEMs was available, most reactions in the EcN model still originated from the iML1515 model, as this model was used as the base for reconstruction. Only ~ 9% of the reactions in the iHM1533 model were added from the other GEMs, many of which contributed only a very limited number of reactions or none at all. Reactions for tRNA charging, for example, were added from other GEMs. While the benefits are modest, the use of multiple models does produce a more complete draft model compared to using only one.

Although this strategy did result in a more complete model, several aspects can still be improved upon. As the comparison of the growth rate of the model to experimental data showed, additional effort is needed to accurately describe EcN’s growth and biomass formation. A new biomass function was defined using BOFdat (Biomass Objective Function from experimental data), a Python package for the generation of species-specific biomass objective functions [62]. Future improvement of the biomass objective function will require EcN specific macromolecular weight fractions and omics datasets in various conditions to get a more accurate description of EcN's growth dynamics. Although the current biomass objective function is not a direct fit to EcN’s growth, it was considered sufficient for this model’s purpose of aiding metabolic engineering and secondary metabolism examination, based on its prediction capacity in the phenotypic microarray and ^13^C fluxomics validation.

In the models used for construction, secondary metabolite biosynthetic pathways were absent or very limited. As a result, the draft model contained only a limited description of these processes. In this work, a more extensive description of the secondary metabolite formation process was defined. Application of the FSEOF algorithm on iHM1533 predicted a total of 219 target reactions to boost production of various secondary metabolites. The fluxes through many of the predicted target reactions changed as the biomass flux was constrained. These target reactions essentially form a set of primary metabolic pathways that enable the switch to secondary metabolites production. As many modular PKS and NRPS products are an important source of antibiotics, there is growing interest in heterologously expressing them in easy to manipulate bacterial hosts. However, *E. coli* has not been a very successful host given the large size of the responsible biosynthetic gene clusters (BGCs) of PKS and NRPS and the high complexity of these modular megaenzymes. EcN is one of the few non-pathogenic *E. coli* strains harbouring an NRPS and two other hybrid PKS-NRPSs, including the 55-kb large colibactin biosynthetic gene cluster. Hence, the iHM1533 may provide a platform to guide future microbial host design for important PKS and NRPS products in *E. coli.*

Model predictions on fluxes and growth phenotypes were compared to experimental data (Biolog and ^13^C fluxomics data). Phenotypic growth data could be predicted with an accuracy of 82.3% and the flux variability analysis validated the ability of predicting internal fluxes. The inability to correctly predict all behaviour is due to the simplification of the model compared to the organism. Multiscale models are gradually developing, in which elements such as enzyme kinetics, 3D structures, or transcriptional regulation are included. For example, Metabolism and macromolecular Expression (ME) models take into account the processes of enzyme synthesis, such as the transcription and translation machinery, folding, and complex formation [63]. Future integration of these processes in the iHM1533 model could improve its predictive power and provide a better understanding of processes such as transcription and translation.

## Conclusion

The genome comparison of EcN to the reference strains showed that EcN shared a total of 1,783 genes with all 55 reference strains, with the highest number of homologous genes shared with *E. coli* CF072, and 196 genes not shared with any other strain. The draft model was manually curated for duplicate reactions, reaction imbalances, energy-generating cycles, and blocked pathways. Comprehensive descriptions of five secondary metabolite biosynthetic pathways were added to the model. Biolog phenotype microarray data was collected to verify the nutrient utilisation. EcN was capable of growth on a wide range of nutrient sources and comparison to a previously published Biolog dataset showed that utilisation of non-carbon nutrients can be dependent upon the carbon source. Verification of model predictions using the phenotype microarray data showed that the model was capable of correctly predicting the growth behaviour of EcN in 82.3% of a total of 230 nutrients. iHM1533 was used to predict gene essentiality of EcN by calculating both single and double lethals, and flux variability analysis of the iHM1533 model was compared to ^13^C fluxomics data and showed accurate prediction of internal fluxes when constraints in optimisation of the biomass formation were relaxed. The final GEM was used to investigate relationships between primary and secondary metabolic pathways and predicted 219 engineering targets from various subsystems for secondary metabolite production. In future studies, iHM1533 can serve as a framework for the integration of various omics data and aid in metabolic engineering applications of EcN.

## Methods

### Reference strains homology analysis

The pipeline for comparison of EcN to the reference strains and construction of the draft model was based on work of Norsigian et al*.* [5], in which a workflow was defined to automatically generate draft multi-strain genome-scale metabolic models based on the high-quality model of a reference strain. Instead in this work, multiple reference strains were used to create one strain-specific model. Fifty-five *Escherichia coli* and *Shigella* strains were chosen as reference strains to represent a larger part of the genomic diversity in the *E. coli* species than each individual model could. All these reference strains have a high-quality genome-scale model available [3]. A high-quality genomic sequence of EcN was available on NCBI (CP022686.1). The reconstruction was performed using Constraint-Based Metabolic Modeling in Python (COBRApy) version 0.16.0 [36].

First, a list of all reference strains with NCBI identifiers was compiled (Additional file 1: Data S1). Annotated genomes of all reference strains and EcN were downloaded from NCBI as genbank files and parsed to generate FASTA files for the protein sequences. A bidirectional protein blast (BLASTp) was run between EcN and the reference strains. Protein sequences with a percentage identity (PID) above an 80% similarity threshold and alignment length of at least 25% were deemed homologous. The best bidirectional hit (BBH) was identified for each EcN gene and a binary orthology matrix was created based on the gene homology (Additional file 1: Data S1). Additionally, bi-directional blast best hits were identified for all *E. coli* K-12 MG1655 genes in comparison to EcN. The heatmap of gene homology of EcN to all reference strains was generated using the binary orthology matrix and visualised with the clustermap function of the seaborn package with default clustering of the genes (Fig. 1D).

### Draft model construction

The MG1655 model (iML1515) was downloaded from the BiGG database in JSON format and reduced by removing all reactions associated with genes that did not have homology to EcN, except S0001, an artificial gene for i.a. spontaneous reactions [64]. Subsequently, models of all other reference strains were taken from the BiGG database. Reactions dependent on EcN homologous genes were added to the reduced model to create the draft EcN model. Gene-protein-reaction (GPR) associations were updated to use EcN gene identifiers after each iteration in this process and the origin of each reaction in the model was saved (Additional file 2: Data S2). Based on the origin and the subsystem of the reactions, a bar plot was created using the barplot function of the seaborn package, to visualise the origin of the reactions in the different subsystems (Fig. 1C).

The draft EcN model was compared to iDK1463 for addition of reactions missing in the draft model. As iDK1463 was based on an older genome sequence, locus tags of the genes differed between the two models. A bidirectional protein blast (BLASTp) was run to correlate the old to the new locus tags. Next, gene names, subsystems, and annotation were updated using the BioCyc and BiGG database [64, 65].

### Curation

The draft model was corrected for duplicate reactions, based on identical reaction formulas and different versions of the same reaction identifier. The BioCyc database and comparison to related strains was used to identify the correct directionality of reactions. Unbound flux cycles found by Memote that involved energy generation were corrected [52]. Mass and charge imbalances were identified and adjusted. Model performance on various carbon sources was compared to literature data. Gap filling was used to identify missing steps that prohibited the model to grow on carbon sources it grows on in vitro on based on literature data. Additionally, gap filling of blocked reactions (based on Memote) identified four pathways that could be corrected by the addition of a reaction from the pangenome of all reference strains. As evidence for the existence of these pathways in EcN was missing in literature and the BioCyc database, no reactions were added. A first analysis of gene essentiality identified two problems in the model; one reaction that should not be present in the model and one wrong annotation of genes. Both were corrected. Similarly, Biolog data showed an inability of EcN of growth on sucrose. As EcN does not have the genes for sucrose catabolism, the reactions SUCtpp, SUCptspp, SUCR, and FFSD were removed from the model [44]. Next, five secondary metabolite biosynthesis pathways were added to the draft model (see next section). Lastly, metabolites and reactions were annotated using the BiGG and KEGG databases [64, 66, 67, 68]. Locus tag, NCBI gene and NCBI protein identifiers were taken from the genbank file of EcN’s genome and annotated to the genes in iHM1533. Both metabolites and genes without reactions were removed from the model.

### Addition of secondary metabolite biosynthetic reactions

We searched for biosynthetic reactions of secondary metabolites at the BiGG database containing curated reactions from many different high-quality GEMs. Only enterobactin and salmochelins biosynthetic pathways were covered in the BiGG database. Most of the GEMs at the BiGG database lack a comprehensive description of complex secondary metabolism. Next, we searched for the biosynthetic reactions described in the MetaCyc database. Comprehensive description of the biosynthesis of enterobactin (ENTBACSYN-PWY), salmochelins (PWY-8023), aerobactin (AEROBACTINSYN-PWY) and colibactin (PWY-8117) were found. The biosynthetic reactions for the yersiniabactin pathway were still represented by a single lumped reaction (PWY-6407). Thus we manually reconstructed yersiniabactin biosynthesis pathways based on chemical steps described in literature [40]. Various metabolites were newly created to constitute a series of intermediate chemical reactions of the pathways (Additional file 2: Data S2). With the help of pathways present in the MetaCyc database and literature, we created corresponding reactions and metabolites (Additional file 2: Data S2). New reactions and metabolite identifiers were assigned, when not present in BiGG or MetaCyc. We further manually curated these pathways for the related gene-protein-reaction rules and added annotations from MetaCyc, MetaNetX and other metabolic databases if available.

### Biomass objective function (BOF) update

BOFdat (Biomass Objective Function from experimental data), a Python package for the generation of species-specific biomass objective functions was used to generate a new biomass objective function (BOF) based on experimental data [62]. BOFdat consists of three independent modules. In the first step, the genome and genbank files (CP022686.1), and transcriptomics data [42] of EcN were used. For the other parameters (macromolecular composition, proteomics, and lipidomics), data from *E. coli* MG1655 was used [62]. The assessment of the maintenance costs was done on a combination of data from MG1655 and EcN, as only two growth conditions were available for EcN [25, 62]. For the second step, inorganic ions and coenzymes were identified in iHM1533 and updated in the product of step one [62]. The third step was skipped, as packages required for execution of this step were no longer functional. Instead, the product from the second step was used to update the existing BOF in the model, originating from the iDK1463 model. The two biomass reaction originating from iML1515 (_core and _WT) were removed from the model.

### Biolog phenotype microarray

EcN was purchased as Mutaflor (Pharma-Zentrale, Germany) and both plasmids (pMut1 & pMut2) were removed. Cells were streaked from glycerol stock on LB agar, grown overnight at 37 °C and restreaked twice. Individual colonies were picked by inoculation loop and suspended in inoculating fluid (IF-0) at an absorbance of 0.37 at 600 nm [69]. This was diluted 1:5 in IF-0 + indicator dye (tetrazolium violet). A 100× dilution of 2 M sodium pyruvate was added to the inoculation fluid for plates PM3 and PM4. The suspended cell solution was inoculated into the appropriate microplates at a volume of 100 µl/well. The PM plates (Biolog Inc., Hayward, CA, USA) were four types of 96-well microplates containing different sources of carbon (PM1 and PM2), nitrogen (PM3), phosphorus and sulfur (PM4). Each PM was tested in triplicate. After inoculation, the microplates were incubated in the OmniLog system for 48 h at 37 °C, with readings every 15 min. The raw kinetic PM data was analysed using DuctApe software which assigns an activity index between 0 and 9 for growth curves. Activity indexes of three and above were considered as growth of EcN (Additional file 5: Data S3). The Biolog data of EcN was compared to the performance of another *E. coli* Nissle strain in literature (Fig. 2B) and the models iHM1533 and iDK1463 (Fig. 2C, Additional file 3: Table S1) [26].

### Comparison phenotype microarray to model

For 319 nutrients of the PM plates, exchange reactions were identified from the BiGG database (Additional file 5: Data S3). Of these, 231 reactions were present in the EcN model. Growth predictions of the model for all available exchange reactions were made on media with defined pyruvate, ammonium, phosphate, sulfate, magnesium and ferric iron, based on the inoculation fluid composition: 2 M sodium pyruvate, 100 mM NaCl, 30 mM triethanolamine HCl (pH 7.1), 5.0 mM NH_4_Cl, 2.0 mM NaH_2_PO_4_, 0.25 mM Na_2_SO_4_, 0.05 mM MgCl_2_, 1.0 mM KCl, and 1.0 μM ferric chloride [70]. The model’s growth was classified as absent when the predicted growth was below 5% of WT growth. A matrix was constructed to visualise the similarity and difference between the model predictions and the Biolog growth data (Fig. 2C). The iDK1463 model was run at the same conditions for comparison (Additional file 5: Data S3). Both models were run a second time with the same media conditions, except 2 M sodium pyruvate was replaced by 2 M sodium succinate and 200 µM ferric citrate. These results were compared to the Biolog data of Kim et al*.* [26] (Additional file 5: Data S3).

In the comparison between iHM1533 and the Biolog data, false-negative nutrients were identified. The gapfilling function of the cobra toolbox was used to predict reactions that would enable growth on these nutrients using a panecoli genome-scale model constructed of the reactions from all reference strains.

### ^13^C flux comparison

To assess the accuracy of predicted fluxes by the iHM1533 model, experimental data describing fluxes in EcN in minimal synthetic medium with 15 mM of glucose or gluconate was used for comparison [25]. The synthetic medium consisted of 48 mM Na_2_HPO_4_, 22 mM KH_2_PO_4_, 9 mM NaCl, 19 mM NH_4_Cl, 2 mM MgSO_4_, 0.1 mM CaCl_2_, 0.1 g/L of thiamine and 15 mM carbon source (glucose or gluconate). The compounds were split up in NH_4_, PO_4_, SO_4_, Mg^2+^, Ca^2+^, Na^+^, K^+^, Cl^−^, thiamine and carbon source to define them as media in silico. All reactions described in the ^13^C experimental data were linked to a reaction in the model.

Flux variability analysis (FVA) and a parsimonious FBA were run (Additional file 7: Data S5). The sensitivity analysis was run with FVA thresholds from 90 to 99%, to evaluate at which sensitivity level the model better matches the experimental data. Resulting datasets were visualised using R Studio (Fig. 3). The python notebook and R script were based on the work by Mol et al*.* (2021) [68].

### Gene essentiality

The essentiality of each gene in iHM1533 in gut microbiome media (Additional file 11: Data S9) [71, 72, 73] for both aerobic and anaerobic conditions was predicted using the function “single_gene_deletion” from the COBRA toolbox (Additional file 8: Data S6). Anaerobic conditions were simulated by setting the oxygen exchange reaction to zero. Gene knockouts in which the growth was less than 5% of WT growth were considered as essential genes. Double lethal simulations were carried out with the “double_gene_deletion” function of the COBRA toolbox. Essential genes were excluded, since double lethals by definition consist of two genes that are not essential on their own.

### Constraint-based flux analysis

Secondary metabolite production levels were predicted using parsimonious FBA using CobraPy. Flux scanning based on an enforced objective flux (FSEOF) algorithm applied by using exchange reactions for each secondary metabolite as objectives to predict reaction amplification targets for secondary metabolite production [55]. The slope of flux variation through various identified targets were calculated against the flux through 5 different secondary metabolite exchange reactions (Additional file 9: Data S7). Since the slope was not constant for all pairs of reactions, we also plotted line plots of flux through secondary metabolites as constrained by an enforced objective on identified target reactions (Fig. 4). In order to understand how the flux through the predicted target reactions varied, we carried out a biomass sensitivity analysis by creating series of model conditions where the biomass reaction was constrained. The flux through the biomass reaction was varied from 0 to 100%, while maintaining the secondary metabolite exchange reaction as an objective (Additional file 10: Data S8). Based on FSEOF predicted target reactions, we selected a total of 20 reactions per secondary metabolite, the 10 highest and 10 lowest slopes of flux variation. These reactions represent top candidates where the fluxes are changing at maximum while transitioning from primary to secondary metabolism. Visualisation of the data was performed with R Studio (Additional file 3: Fig S4–S8). The R script was based on work by Mol et al*.* [74].

### Memote

To compare the performance of iHM1533, iDK1463, and iML1515, all three models were run on the Memote web application and scores were compared (Additional file 3: Fig S3) [52].

## Supplementary Information

**Additional file 1.** Data S1.

**Additional file 2.** Data S2.

**Additional file 3.** Supplementary figures and tables.

**Additional file 4.** iHM1533 model.

**Additional file 5.** Data S3.

**Additional file 6.** Data S4.

**Additional file 7.** Data S5.

**Additional file 8.** Data S6.

**Additional file 9.** Data S7.

**Additional file 10.** Data S8.

**Additional file 11.** Data S9.

## Data Availability

The strain name, accession number of the reference genome and reference model for each strain used in this work: *Escherichia coli* Nissle 1917, CP022686.1, iDK1463; *Escherichia coli* LF82, CU651637, iLF82_1304; *Escherichia coli* O83:H1 str. NRG 857C, CP001855, iNRG857_1313; *Escherichia coli* UM146, CP002167, iUMN146_1321; *Escherichia coli* APEC O1, CP000468, iAPECO1_1312; *Escherichia coli* ATCC 8739, CP000946, iEcolC_1368; *Escherichia coli* B str. REL606, CP000819, iECB_1328; *Escherichia coli* BL21(DE3) BL21-Gold(DE3)pLysS AG, CP001665, iECBD_1354; *Escherichia coli* BL21(DE3) AM946981, AM946981, iB21_1397; *Escherichia coli* BL21(DE3) CP001509, CP001509, iECD_1391; *Escherichia coli* BW2952, CP001396, iBWG_1329; *Escherichia coli* DH1, CP001637, iEcDH1_1363; *Escherichia coli* DH1 ME8569, AP012030, iECDH1ME8569_1439; *Escherichia coli* ED1a, CU928162, iECED1_1282; *Escherichia coli* HS, CP000802, iEcHS_1320; *Escherichia coli* IAI1, CU928160, iECIAI1_1343; *Escherichia coli* KO11FL, CP002516, iEKO11_1354; *Escherichia coli* SE11, AP009240, iECSE_1348; *Escherichia coli* SE15, AP009378, iECSF_1327; *Escherichia coli* str. K-12 substr. DH10B, CP000948, iECDH10B_1368; *Escherichia coli* str. K-12 substr. MG1655, U00096, iJO1366; *Escherichia coli* str. K-12 substr. W3110, AP009048, iY75_1357; *Escherichia coli* W CP002185, CP002185, iECW_1372; *Escherichia coli* W, CP002967, iWFL_1372; *Escherichia coli* 042, FN554766, iEC042_1314; *Escherichia coli* 55,989, CU928145, iEC55989_1330; *Escherichia coli* O103:H2 str. 12,009, AP010958, iECO103_1326; *Escherichia coli* O111:H- str. 11,128, AP010960, iECO111_1330; *Escherichia coli* O157:H7 str. EC4115, CP001164, iECH74115_1262; *Escherichia coli* O157:H7 EDL933, AE005174, iZ_1308; *Escherichia coli* O157:H7 str. Sakai, BA000007, iECs_1301; *Escherichia coli* O157:H7 str. TW14359, CP001368, iECSP_1301; *Escherichia coli* O26:H11 str. 11,368, AP010953, iECO26_1355; *Escherichia coli* O55:H7 str. CB9615, CP001846, iG2583_1286; *Escherichia coli* SMS-3-5, CP000970, iEcSMS35_1347; *Escherichia coli* O127:H6 str. E2348/69, FM180568, iE2348C_1286; *Escherichia coli* E24377A, CP000800, iEcE24377_1341; *Escherichia coli* ETEC H10407, FN649414, iETEC_1333; *Escherichia coli* UMNK88, CP002729, iUMNK88_1353; *Escherichia coli* IHE3034, CP001969, iECOK1_1307; *Escherichia coli* S88, CU928161, iECS88_1305; *Escherichia coli* 536, CP000247, iECP_1309; *Escherichia coli* ABU 83972, CP001671, iECABU_c1320; *Escherichia coli* CFT073, AE014075, ic_1306; *Escherichia coli* IAI39, CU928164, iECIAI39_1322; *Escherichia coli* NA114, CP002797, iECNA114_1301; *Escherichia coli* UMN026, CU928163, iECUMN_1333; *Escherichia coli* UTI89, CP000243, iUTI89_1310; *Shigella boydii* CDC 3083-94, CP001063, iSbBS512_1146; *Shigella boydii* Sb227, CP000036, iSBO_1134; *Shigella dysenteriae* Sd197, CP000034, iSDY_1059; *Shigella flexneri* 2,002,017, CP001383, iSFxv_1172; *Shigella flexneri* 2a str. 2457T, AE014073, iS_1188; *Shigella flexneri* 2a str. 301, AE005674, iSF_1195; *Shigella flexneri* 5 str. 8401, CP000266, iSFV_1184; *Shigella sonnei* Ss046, CP000038, iSSON_1240. The jupyter notebooks, R scripts and data used to reconstruct, validate, and analyse the iHM1533 model are available at GitHub link https://github.com/maxvthof/EcN_model.

## Abbreviations

BOFdat: Biomass objective function from experimental data
EcN: *Escherichia coli* Nissle 1917
ED: Entner-Doudoroff pathways
ExPEC: Extraintestinal pathogenic *E. coli*
FBA: Flux balance analysis
FSEOF: Flux scanning with enforced objective function
FVA: Flux variability analysis
GAM: Growth-associated maintenance energy
GEI: Genomic island
GEM: Genome-scale metabolic model
InPEC: Intestinal pathogenic *E. coli*
ME model: Metabolism and macromolecular Expression model
NGAM: Non-growth-associated maintenance energy
NRPS: Non-ribosomal peptide synthase
pFBA: Parsimonious flux balance analysis
PID: Percentage identity
PKS: Polyketide synthase
PPP: Pentose phosphate pathway
TCA: Tricarboxylic acid cycle
UPEC: Uropathogenic *E. coli*
