# Supplementary material for: High-quality genome-scale metabolic network reconstruction of probiotic bacterium Escherichia coli Nissle 1917
Source: BMC Bioinformatics. 2022 Dec 30;23:566. doi: 10.1186/s12859-022-05108-9 (PMC9801561; doi:10.1186/s12859-022-05108-9)
Supplement: Supplementary file 3 — Additional file 3. Supplementary figures and tables. [file 12859_2022_5108_MOESM3_ESM.pdf]

Additional file 3: Supplementary Information

High-quality genome-scale metabolic network reconstruction of probiotic bacterium *Escherichia coli* Nissle 1917

Max van ‘t Hof[1], Omkar S. Mohite[1], Jonathan M. Monk[2], Tilmann Weber[1], Bernhard O. Palsson[1,2], Morten O. A. Sommer[1#]

[1]The Novo Nordisk Foundation Center for Biosustainability, Technical University of Denmark, Kongens Lyngby 2800, Denmark.
[2]Department of Bioengineering, University of California San Diego, La Jolla, CA 92093, USA.

[#]**Correspondence:** msom@bio.dtu.dk

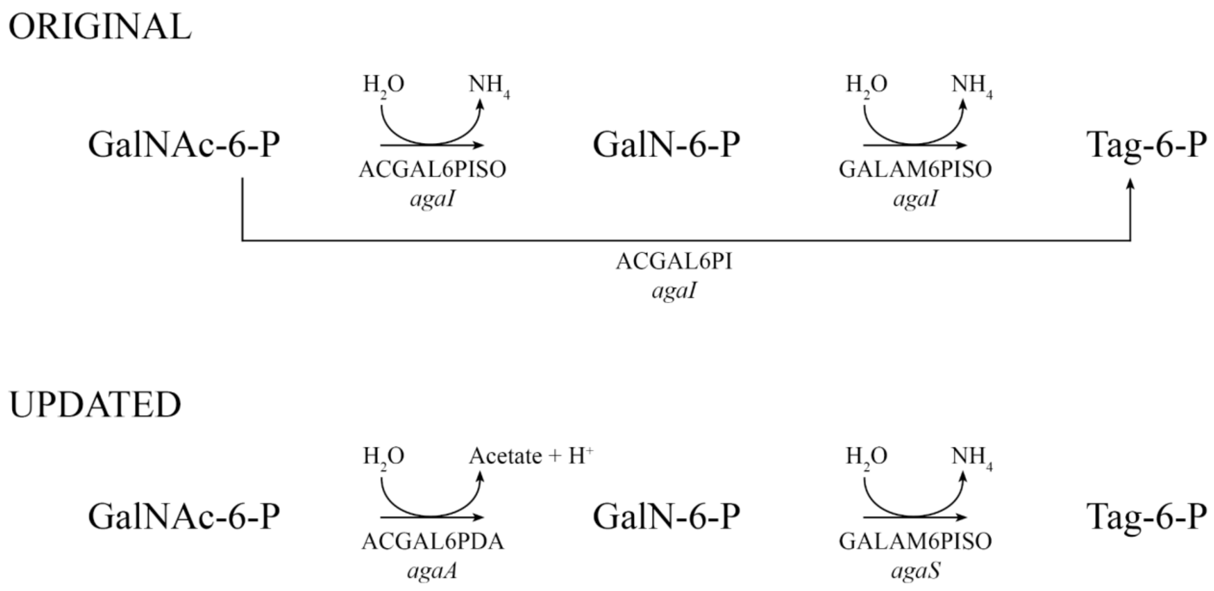

Figure S1. N-acetyl-D-galactosamine-6-phosphate conversion to D-tagatose-6-phosphate

During manual curation a mass imbalance was observed of two reactions (ACGAL6PI & ACGAL6PISO) involving the metabolite N-acetyl-D-galactosamine-6-phosphate (GalNac-6-P). In nature, the conversion of this compound (GalNAc-6-P to Tag-6-P) is performed in two steps: deacetylation of GalNAc-6-P to GalN-6-P and deamination/isomerisation of GalN-6-P to Tag-6-P [35]. The model contained both a two-step conversion (ACGAL6PISO & GALAM6PISO) and a reaction that directly converted GalNac-6-P to Tag-6-P (ACGAL6PI) without any by-products. Additionally, ACGAL6PISO had ammonium as a by-product instead of acetate. Both original reactions (ACGAL6PI & ACGAL6PISO) were removed. Instead a new reaction was added for the deacetylation step (ACGAL6PDA), while the deamination/isomerase was already present in the model (GALAM6PISO). Genes associated to the reactions were updated to *agaA* and *agaS*, respectively [75].

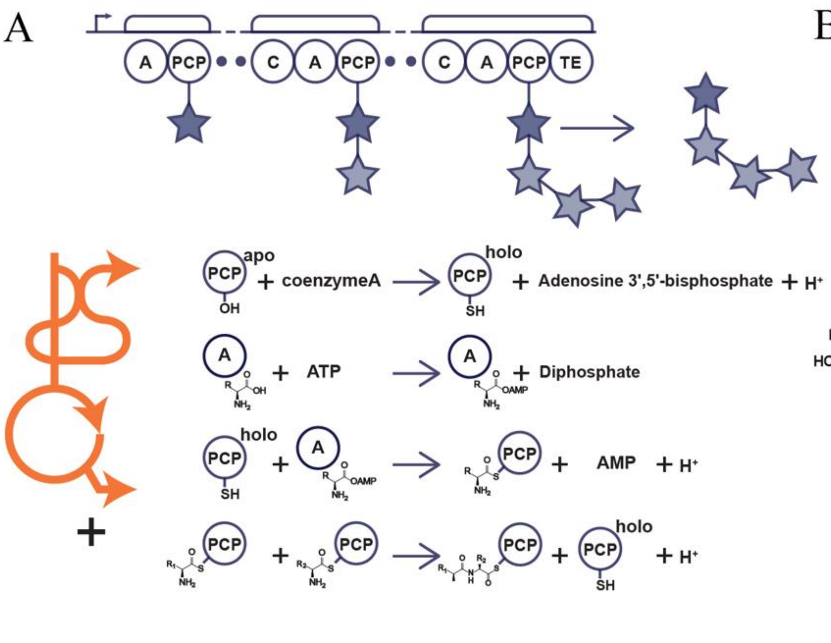

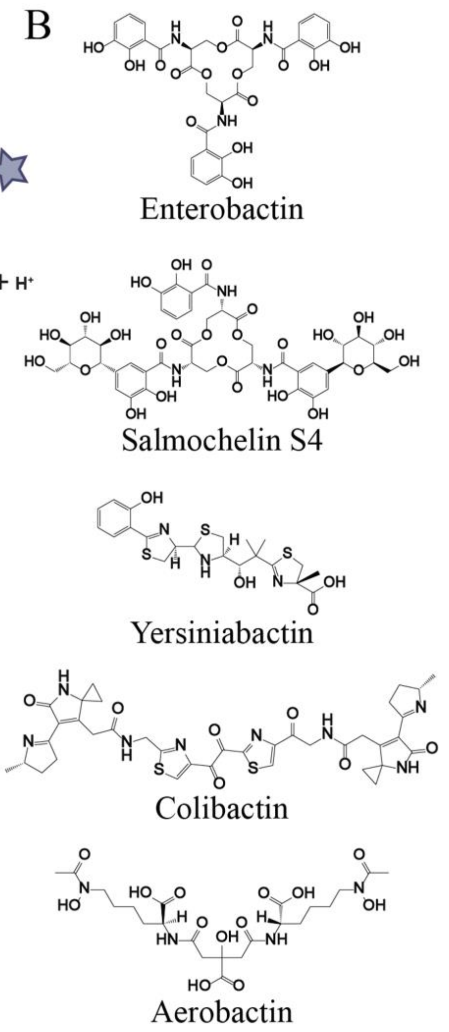

C

Secondary metabolite pathway	Reactions	Metabolites
Enterobactin	31	8
Salmochelin	25	0
Yersiniabactin	54	46
Colibactin	55	53
Aerobactin	12	3

Figure S2. Secondary metabolite biosynthesis pathways in *E. coli* Nissle 1917

A. Extending GEMs with detailed comprehensive steps in secondary metabolite biosynthetic pathway. Example of a typical NRPS assembly line biosynthesis and some of the biochemical steps involved: priming of the peptidyl carrier protein (PCP) domain, activation of an amino acid in the adenylation (A) domain, transfer of the activated amino acid to the PCP domain, and condensation of the molecules on two adjacent modules by the condensation (C) domain. The thioesterase (TE) domain is located in the termination module and catalyzes the release from the peptide. B. Diverse secondary metabolites produced by *E. coli* Nissle 1917 including enterobactin, salmochelin S4 (glucosylated enterobactin), yersiniabactin, aerobactin, and colibactin. C. Number of reactions present in the manually curated secondary metabolite pathways and number of newly added metabolites per pathway.

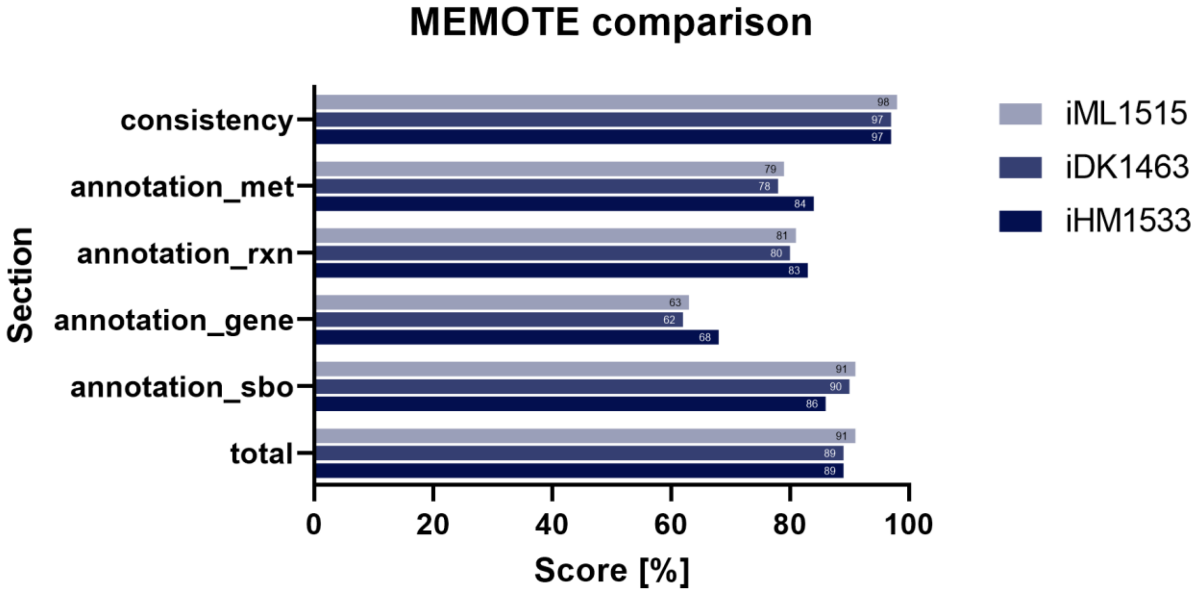

Figure S3. Memote comparison of iML1515, iDK1463 & iHM1533

The Memote web application, a standard in the community for metabolic model testing, was used to compare the consistency, and annotation of metabolites (met), reactions (rxn), genes (gene), and SBO terms (sbo) of iML1515, iDK1463, and iHM1533. The lower score of iHM1533 in consistency was largely due to mass and charge imbalances as a result of the peptidyl carrier protein (PCP) domain being a part of secondary metabolite formulas as a rest group (R).

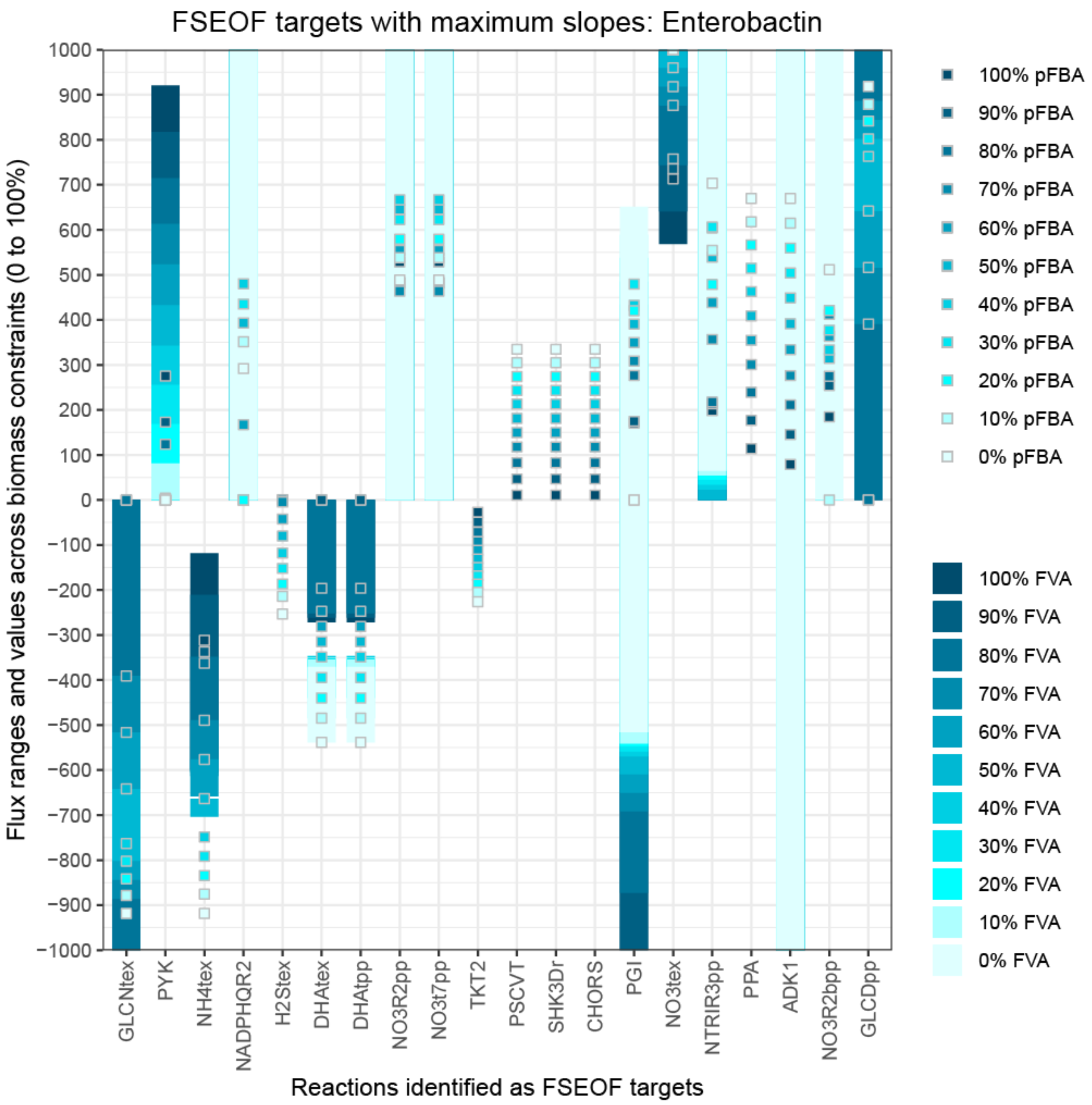

Figure S4. Predicted fluxes thorough key FSEOF targets for enterobactin calculated across various biomass constraints

The x-axis includes FSEOF predicted target reactions with 10 lowest (negative) and 10 highest (positive) slopes. The reactions with highest positive slopes in FSEOF represents targets that positively influence secondary metabolites production, whereas negative slopes represent reduction in secondary metabolite production. The colour range represents series of models with constrained flux from 0% (only secondary metabolite production) to 100% (only biomass production) in the biomass reaction. The bars represent allowed flux range calculated using FVA at each of the constrained models, whereas the points represent the optimal flux value of the target reaction calculated using pFBA.

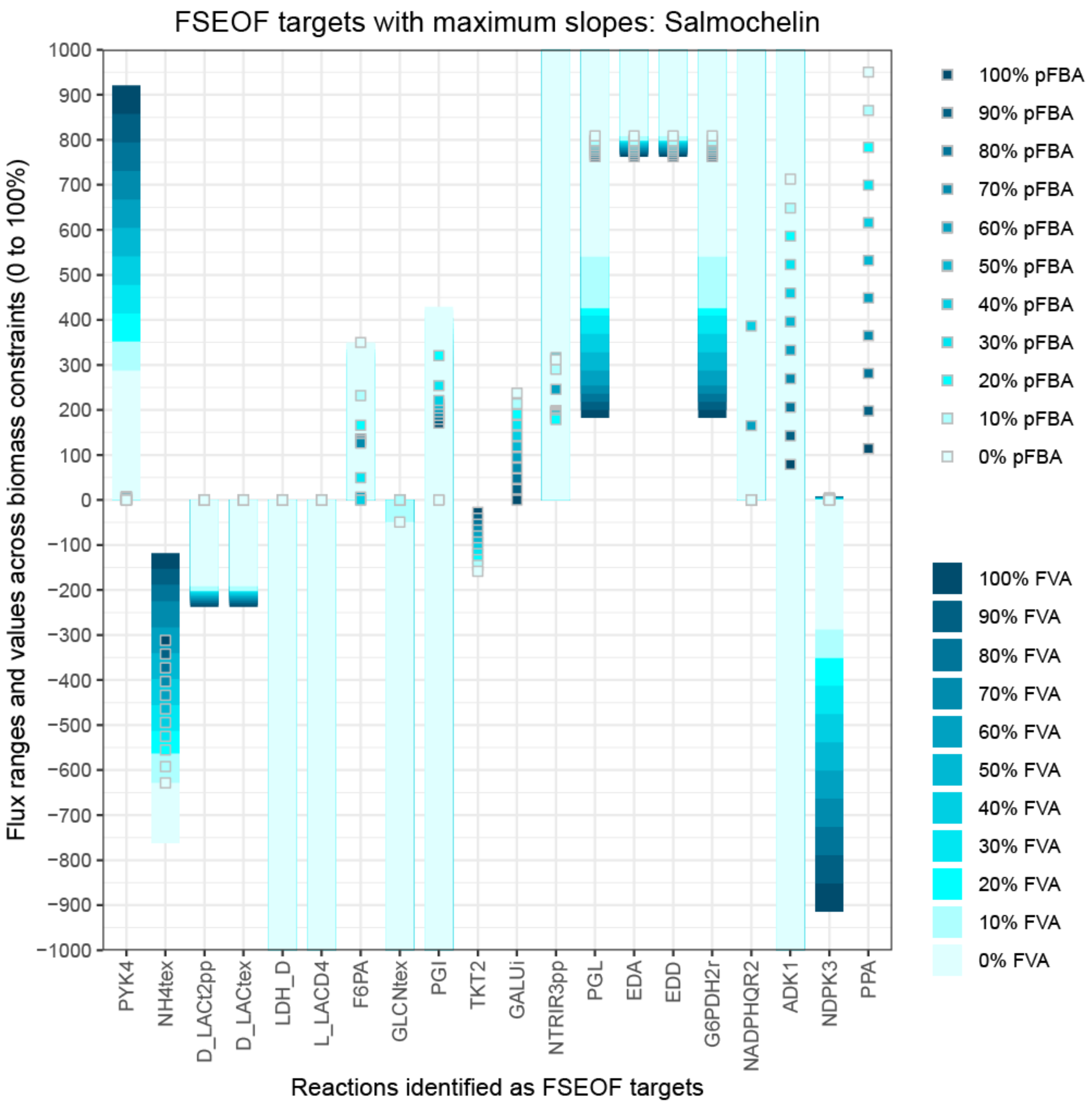

Figure S5. Predicted fluxes thorough key FSEOF targets for salmochelin calculated across various biomass constraints

The x-axis includes FSEOF predicted target reactions with 10 lowest (negative) and 10 highest (positive) slopes. The reactions with highest positive slopes in FSEOF represents targets that positively influence secondary metabolites production, whereas negative slopes represent reduction in secondary metabolite production. The colour range represents series of models with constrained flux from 0% (only secondary metabolite production) to 100% (only biomass production) in the biomass reaction. The bars represent allowed flux range calculated using FVA at each of the constrained models, whereas the points represent the optimal flux value of the target reaction calculated using pFBA.

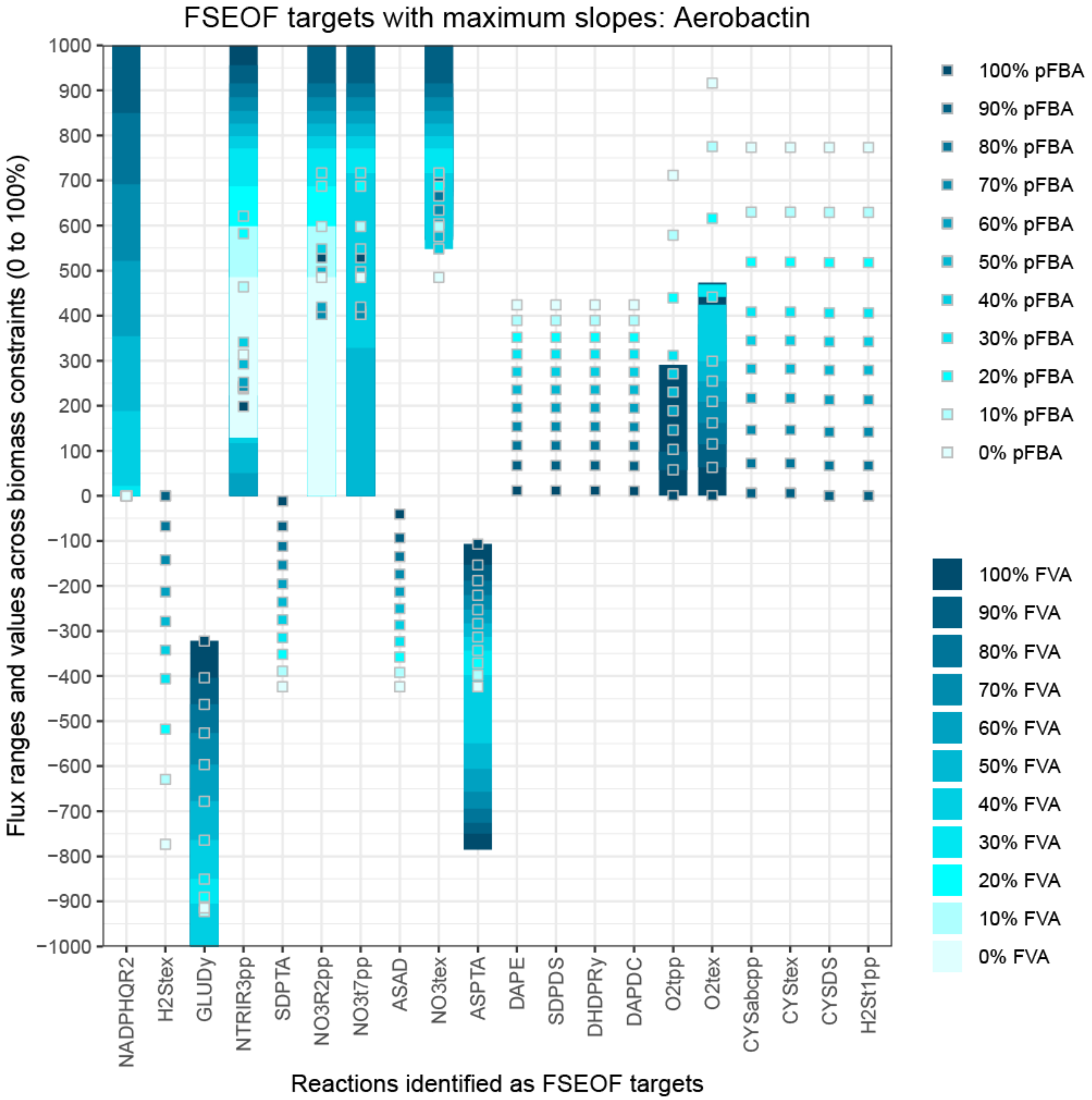

Figure S6. Predicted fluxes thorough key FSEOF targets for aerobactin calculated across various biomass constraints

The x-axis includes FSEOF predicted target reactions with 10 lowest (negative) and 10 highest (positive) slopes. The reactions with highest positive slopes in FSEOF represents targets that positively influence secondary metabolites production, whereas negative slopes represent reduction in secondary metabolite production. The colour range represents series of models with constrained flux from 0% (only secondary metabolite production) to 100% (only biomass production) in the biomass reaction. The bars represent allowed flux range calculated using FVA at each of the constrained models, whereas the points represent the optimal flux value of the target reaction calculated using pFBA.

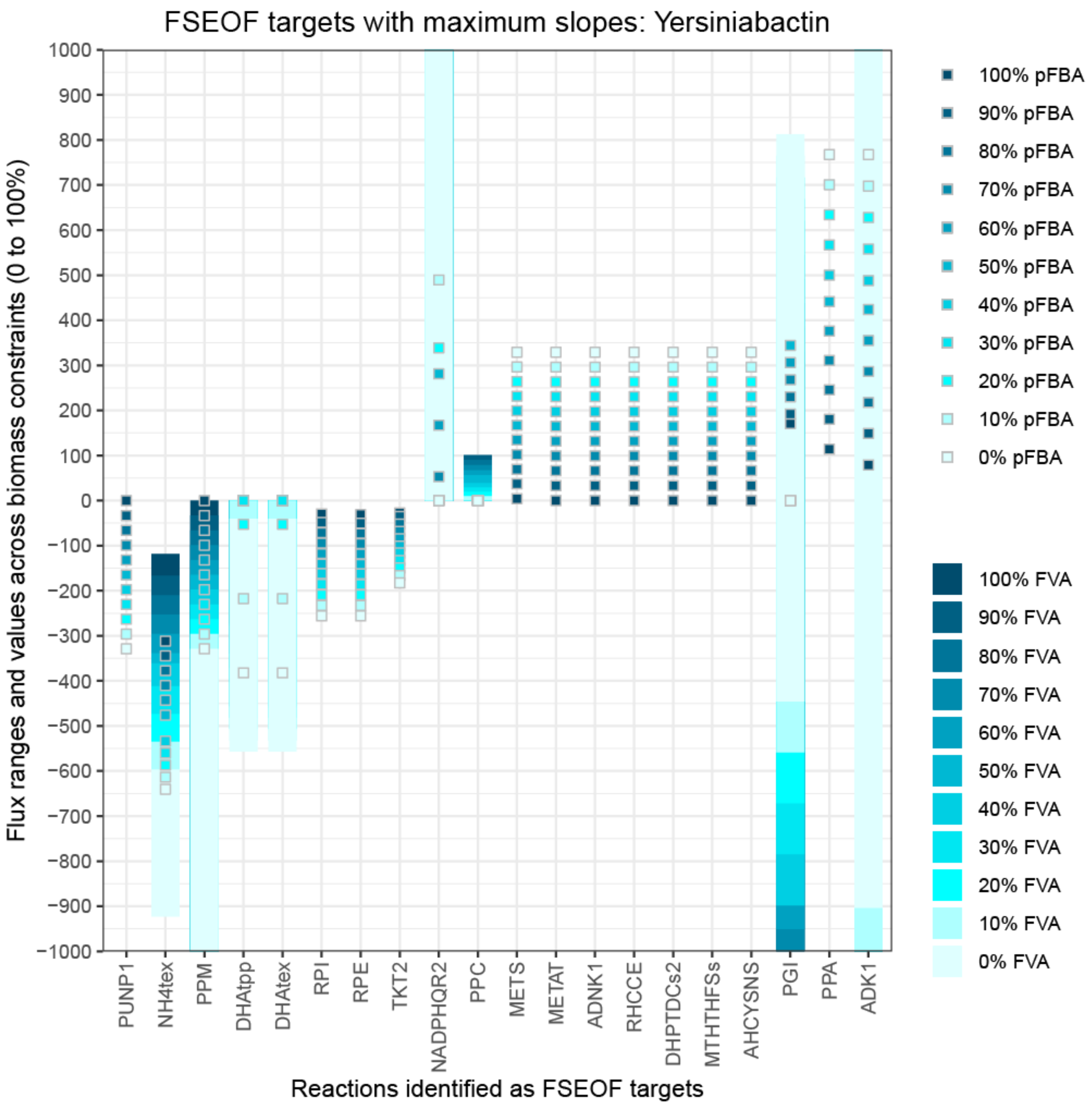

Figure S7. Predicted fluxes thorough key FSEOF targets for yersiniabactin calculated across various biomass constraints

The x-axis includes FSEOF predicted target reactions with 10 lowest (negative) and 10 highest (positive) slopes. The reactions with highest positive slopes in FSEOF represents targets that positively influence secondary metabolites production, whereas negative slopes represent reduction in secondary metabolite production. The colour range represents series of models with constrained flux from 0% (only secondary metabolite production) to 100% (only biomass production) in the biomass reaction. The bars represent allowed flux range calculated using FVA at each of the constrained models, whereas the points represent the optimal flux value of the target reaction calculated using pFBA.

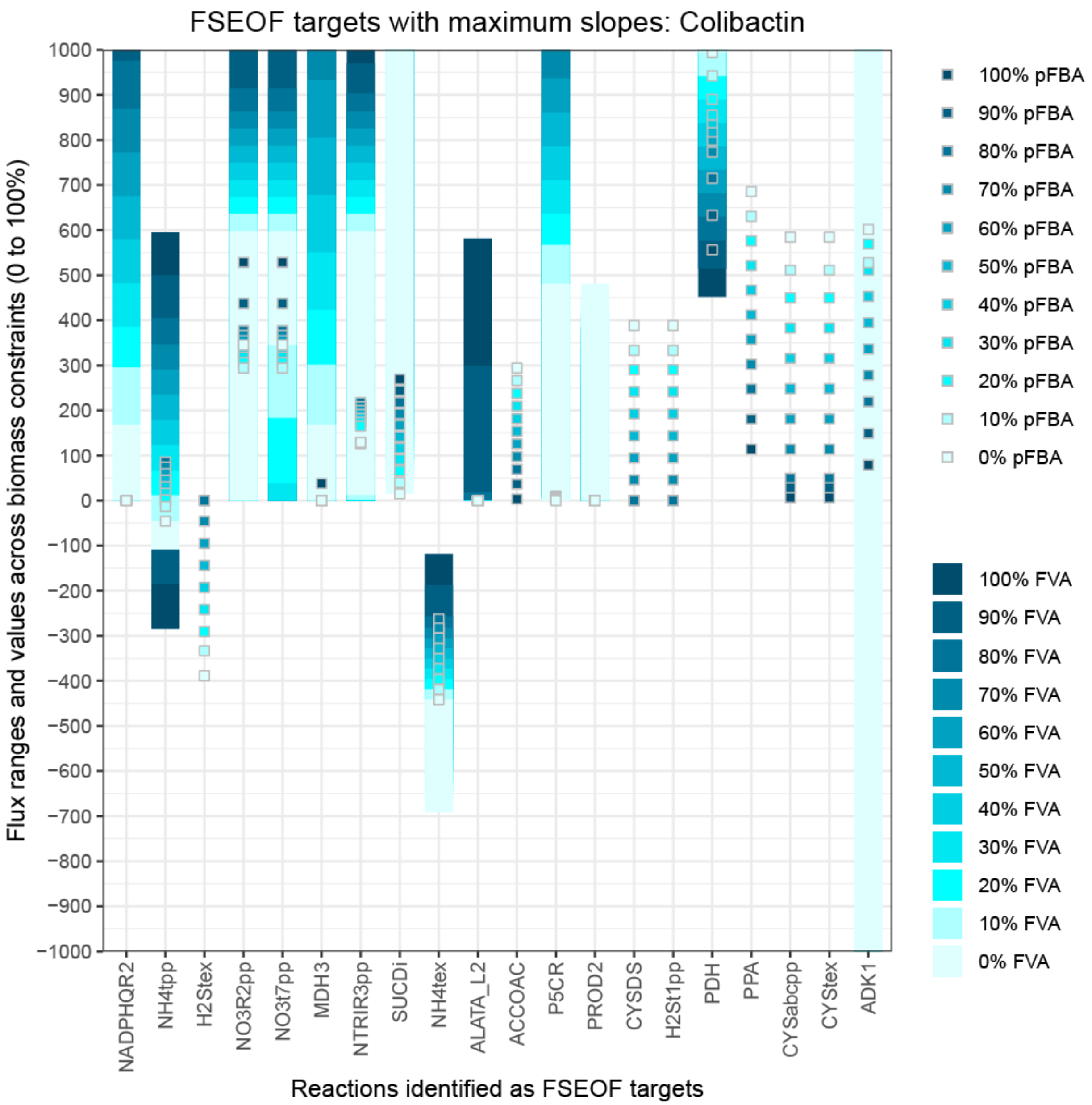

Figure S8. Predicted fluxes thorough key FSEOF targets for colibactin calculated across various biomass constraints

The x-axis includes FSEOF predicted target reactions with 10 lowest (negative) and 10 highest (positive) slopes. The reactions with highest positive slopes in FSEOF represents targets that positively influence secondary metabolites production, whereas negative slopes represent reduction in secondary metabolite production. The colour range represents series of models with constrained flux from 0% (only secondary metabolite production) to 100% (only biomass production) in the biomass reaction. The bars represent allowed flux range calculated using FVA at each of the constrained models, whereas the points represent the optimal flux value of the target reaction calculated using pFBA.

Table S1. Performance of EcN models iDK1463 and iHM1533 on carbon sources

Carbon	Model	TP	FP	FN	TN	Score
Pyruvate	iDK1463	121	21	21	68	81.8%
	iHM1533	122	21	20	68	82.3%
Succinate	iDK1463	130	12	24	65	81.8%
	iHM1533	131	12	23	65	82.3%

Prediction of growth on various nutrients by the EcN models iDK1463 and iHM1533 was compared to Biolog data. This was done with two different carbon sources for PM03 and PM04; 2M sodium pyruvate for the data set generated in this work and 2M sodium succinate/200 µM ferric citrate for the data set. Four outcomes were possible: The model correctly predicted growth (True positive, TP) or no growth (True negative, TN). Alternatively, the model false predicted growth (False positive, FP), or did not predict growth, while EcN was capable of growth on this nutrient (False negative, FN). The score represents the number of correct predictions divided by the total number of predictions.

Table S2. Predicted engineering targets by FSEOF for secondary metabolite production

Subsystem	Yersiniabactin	Colibactin	Enterobactin	Salmochelin	Aerobactin
Transport, Outer Membrane	8	10	10	8	16
Nucleotide Salvage Pathway	11	10	9	8	6
Pentose Phosphate Pathway	9	9	7	7	7
Transport, Inner Membrane	8	8	7	5	11
Alternate Carbon Metabolism	8	8	7	3	6
Cofactor and Prosthetic Group Biosynthesis	4	5	4	7	10
Citric Acid Cycle	8	1	8	8	2
Membrane Lipid Metabolism	1	25	0	0	0
Inorganic Ion Transport and Metabolism	4	5	5	7	4
Oxidative Phosphorylation	3	4	5	6	6
Glycolysis/Gluconeogenesis	5	3	4	3	7
Tyrosine, Tryptophan, and Phenylalanine Metabolism	7	0	7	7	0
Pyruvate Metabolism	3	4	3	6	5
Glycine and Serine Metabolism	4	4	3	3	2
Threonine and Lysine Metabolism	0	3	0	0	9

Methionine Metabolism	6	5	0	0	0
Nitrogen Metabolism	1	2	3	1	3
Anaplerotic Reactions	2	2	2	1	3
Valine, Leucine, and Isoleucine Metabolism	2	2	2	2	0
Unassigned	1	3	1	1	1
Folate Metabolism	2	2	0	0	2
Purine and Pyrimidine Biosynthesis	1	1	1	1	1
Alanine and Aspartate Metabolism	0	2	0	0	2
Glutamate Metabolism	0	1	1	0	1
Cysteine Metabolism	0	1	1	0	1
Arginine and Proline Metabolism	0	3	0	0	0
Cell Envelope Biosynthesis	0	0	0	1	0
Transport, Outer Membrane	8	10	10	8	16

Number of the FSEOF predicted reaction targets per subsystem for secondary metabolite biosynthesis. Several reactions from transport metabolism of common metabolites such as water, CO_2, and energy storage metabolites such as NADH were ignored.
